# What do we know about canine osteosarcoma treatment? – review

**DOI:** 10.1007/s11259-014-9623-0

**Published:** 2014-11-26

**Authors:** M. Szewczyk, R. Lechowski, K. Zabielska

**Affiliations:** Department of Small Animal Diseases with Clinic, Faculty of Veterinary Medicine, Warsaw University of Life Sciences, Nowoursynowska 159c, 02-787 Warsaw, Poland

**Keywords:** Osteosarcoma, Dogs, Treatment, Chemotherapy

## Abstract

Osteosarcoma (OSA) is the most common type of bone tumors in dogs, which has high metastasis ability. 80 % of dogs with OSA die due to lung metastasis. As a result its treatment is a challenge for veterinary practitioners. The authors discuss the etiology, pathogenesis and the possible risk factors of OSA. The article focuses on literature review and the study of recent advances in OSA treatment. The authors describe therapies which have significantly prolonged the lives of dogs, as well as those that have proven to be ineffective. Advantages and disadvantages of limb amputation and limb-sparing surgery have been described. Authors present also the results of both single agent’s therapies with the most commonly used drugs as cisplatin, carboplatin and doxorubicin and compare them to the results obtained using combined chemotherapy. The use of nanotechnology as a new approach in OSA treatment in order to avoid multidrug resistance and reduce negative side effects of cytostatic drugs is presented. The main reasons of the therapies failure are also provided in this article.

## Introduction

**Fig 1:**
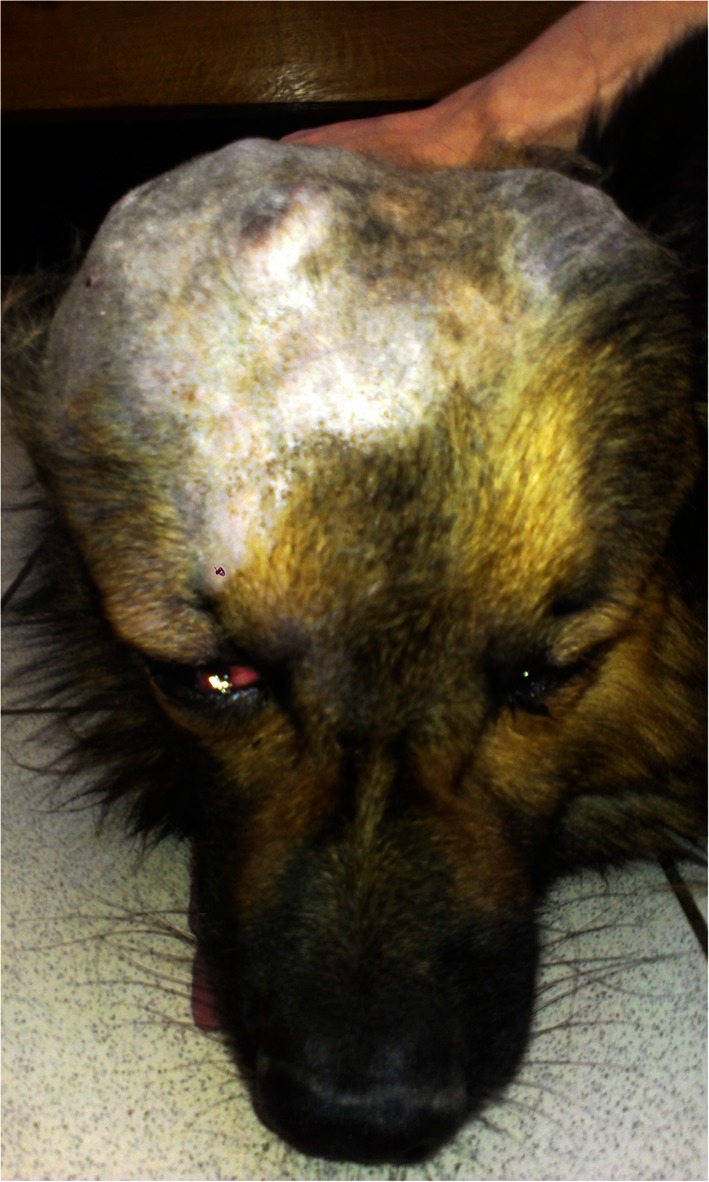
A 10-year old mixed breed dog with skull osteosarcoma

Osteosarcoma (OSA) is the most common bone tumor in dogs (more than 80 % of malignant bone tumors). It mainly occurs in large and giant breeds such as: Rottweiler, German Shepherd, Boxer, Doberman Pinscher, Irish Setter (Spodnick et al. 1992; Berg 1996; Cavalcanti et al. 2004; Morello et al. 2011). It most often appears in middle age dogs (between 6 and 10 years old) (Thompson and Pool 2002; Morello et al. 2011), but it has also been reported in 1–2 year old dogs (Brodey 1979). The topographic location is the appendicular skeleton (64 % of cases), the axial skeleton (28, 5 %) (ribs and skull) (Fig. 1) and the extraskeletal muscles (7, 5 %) (Calvacanti et al. 2004; Trost et al. 2012). Appendicular OSA appears more often in forelimbs than in hindlimbs, whereas extraskeletal OSA develops primarily in visceral organs (adrenal gland, eye, gastric ligament, ileum, kidney, liver, spleen, testicle and vagina) (Langenbach et al. 1998).

## Risk factors

The ethiopathogenesis of OSA is unknown, but various predisposing factors (sex, body weight) may lead to its development. Dogs with a body weight above 40 kg are more predisposed than smaller dogs (Bergman et al. 1996). Most of the studies indicate that this neoplasm tends to affect males more often than females (Brodey and Abt 1976; Jongeward 1985; Pool 1990; Selverajah and Kirpenstein 2010). However, according to certain reports also females are predisposed. Cooley et al. (2002) indicate that there may be a correlation between castration and a higher risk of tumor development. Male and female dogs that underwent gonadectomy before 1 year of age had a one in four lifetime risk for bone sarcoma and they were significantly more likely to develop bone sarcoma than dogs that were sexually intact.

The location of a neoplasm increases the hazard of metastasis and mortality. Tumors localized at distal radius are associated with a lower hazard of metastasis, while tumors localized at proximal humerus and distal femur or proximal tibia have a high metastasis ability, which in turn results in a significant increase in mortality (Schmidt et al. 2013). Clinical signs depend on the location of primary tumors. In appendicular OSA, the typical clinical signs are: lameness (with or without noticeable pain) and local swelling at the tumor site, which is usually a consequence of the tumor’s extension into the surrounding soft tissues (Brodey 1979; Jongeward 1985).

## Diagnosis

**Fig 2:**
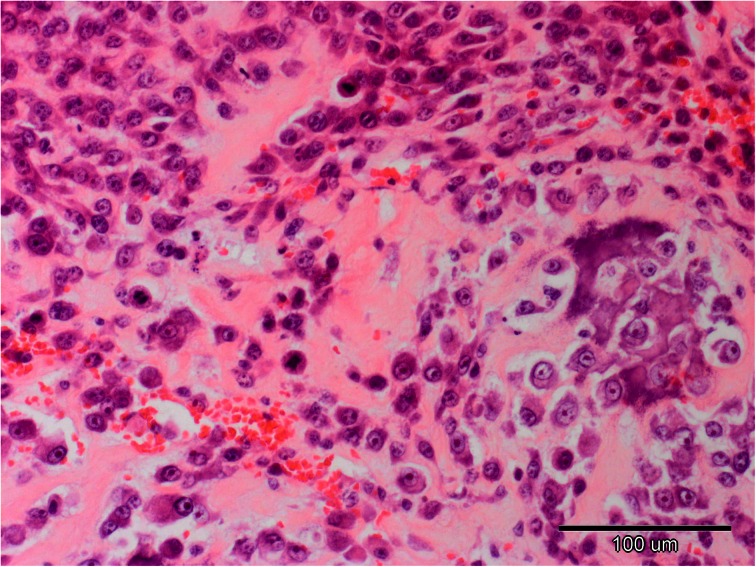
Canine osteosarcoma stained with hematoxylin-eosin method, original magnification 200x

Diagnosis is based on physical examination, radiography of the lesion and fine needle biopsy performed in order to identify the type of tumor (Fig. 2) (Mehl et al. 2001; Thompson and Pool 2002). X-Ray of the chest is recommended as an additional test because of high metastatic risk. A blood test, CT scan or MRI should be performed if limb-sparing surgery is considered. According to the TNM system (T-tumor, N-lymph node, M-metastasis) it is possible to differentiate 3 stages of the disease. Stage I includes low-grade (G1) lesion without evidence of metastasis (M0); stage II includes high-grade (G2) lesion without metastasis (M0); and stage III is lesion with metastasis disease (M1). Irrespectively of the histologic grade, the stages I and II are subdivided by the anatomic setting for two groups (A, B). Group A is intracompartmental (tumor has remained within the bone) (T1), while group B is extracompartmental (tumor has extended beyond the bone into other nearby structures) (T2). Most dogs are diagnosed with stage IIB OSA (Withrow et al. 2013).

## Treatment

Treatment includes: surgery (limb amputation or limb-sparing surgery), radiotherapy and chemotherapy (Boston et al. 2006). Amputation is a first-line procedure, which increases survival, brings pain relief, thereby delays euthanasia (Spodnick et al. 1992; Thompson and Pool 2002; Selvarajah and Kirpenstejin 2010). It completely removes the primary tumor, decreases the risk of postoperative complications, shortens the time of anesthesia and decreases the expense in comparison with the limb-sparing procedure (Jehn et al. 2007).

### Surgical treatment

Surgery (either limb amputation or limb-sparing surgery) is the first method of treatment. Limb-sparing surgery is an alternative method to limb amputation. It is a surgical procedure in which the bone tumor is resected without limb amputation by reconstructing the excised bone segment, thus preserving the limb. Covey et al. (2014) insisted that internal fixation when following stereotactic radiosurgery may be a viable alternative to limb amputation. The bone can be reconstructed with an endoprothesis (metal implant) or cortical allograft. It has been proven that the type of implant has no influence on the construct failure or on the postoperative infection (Liptak et al. 2006). Circular external fixators are also commonly used. Limb function is preserved in over 80 % of dogs following limb-sparing surgery, however, complications such as infections (in 30–50 % of patients) or implant failure (20–40 %) are relatively common. Moreover, tumor recurrence appears in 15–25 % of cases. As a result this technique is recommended for dogs with compromising neurologic or orthopedic problems, or it can be favorable for owners who refuse to perform limb amputation (Straw and Withrow 1996; MacDonald and Schiller 2010).

However, dogs treated with surgery alone have a short median survival time. Authors from North Carolina State University showed that 72, 5 % of dogs with appendicular OSA treated by amputation alone died or were euthanized because of metastases after 138 days (from diagnosis) (Spodnick et al. 1992). As OSA are highly aggressive tumors, micrometastasies occur in over 90 % of dogs. (O’Brien et al. 1993; MacEwen and Kurzman 1996; Selvarajah and Kirpensteijn 2010). Usually metastases occur in the lungs and bones, but they may be also found in regional lymph nodes or internal organs (spleen, liver) (Ogilvie et al. 1993). The information in medical records collected between 1986 and 2003 suggests that dogs with regional lymph nodes metastasies live shorter (48 days) than dogs without metastasies in lymph nodes (318 days) (Hillers et al. 2005). Outcomes gathered during 19 years showed staging results. Dogs with stage III OSA had poor prognosis. The median survival time was 76 days (Boston et al. 2006).

### Chemotherapy

As a result, various studies have been performed to assess if the survival of dogs with OSA can be prolonged by adjuvant therapy (Berg 1996; Moore et al. 2007; Phillips et al. 2009; Skorupski et al. 2013). Latest reports show efficiency of a few cytostatic drugs. Most commonly used cytostatics are: carboplatin, cisplatin, and doxorubicin.

#### Carboplatin

Authors from Veterinary Specialty Hospital of San Diego compared the median survival time of 48 dogs with appendicular OSA after receiving single-agent carboplatin (300 mg/m(2) IV q21d for 4 cycles) following amputation to amputation alone. Dogs treated with adjunctive therapy had a prolonged median survival time (307 days) in comparison to those after surgery alone (approximately 138 days) (Bergman et al. 1996; Phillips et al. 2009). Saam et al. (Saam et al. 2011) compared the outcomes collected between 1996 and 2006 from 65 dogs with adjuvant carboplatin-treated OSA using a similar protocol and showed that carboplatin administration is well tolerated and median survival time is similar to those treated with other chemotherapeutics (doxorubicin or cisplatin). Interesting studies were performed by Simcock et al. (Simcock et al. 2012) who evaluate the adverse effects and survival times in 17 dogs that had OSA treated with a single subcutaneous infusion of carboplatin (dosage 300 mg/m2 infused over a 3, 5 or 7 day period) as an adjunctive therapy following the amputation of the affected limb. The results were comparable to those of previous reports, where carboplatin was given intravenously. However, it should be noticed that there were only 17 dogs included in the study; therefore further studies should be performed in order to confirm these results.

#### Cisplatin

Some authors indicate a significantly longer median survival time for dogs with appendicular OSA treated with cisplatin as an adjuvant therapy to amputation or limb-sparing surgery (322 days), than with surgery alone (138 days) (Kraegel et al. 1991; Berg et al. 1992). Similar results were published by Straw et al. (Straw et al. 1991), who showed that 71 dogs treated with cisplatin as an adjuvant therapy had significantly longer median survival times than dogs with no chemotherapy. However, in the treated group 73, 4 % of dogs were euthanatized because of the problem related to metastases, which was significantly higher than in the group of dogs with amputation alone. The results obtained indicate that cisplatin treatment is effective, but it does not inhibit metastases. Another research of Hahn et al. (1996) assessed the effectiveness of cisplatin administrated intramedullary. The survey concerned 4 dogs with OSA to such an advanced stage that they were not eligible for an amputation or limb-sparing surgery. One out of four dogs undertaken with this treatment was found to be tumor-free, another one had partial remission of local neoplasm and in two dogs the disease had evolved. However, there were only 4 dogs included in the study, which weakens the reliability of the results. Further studies including many more animals should be performed.

#### Doxorubicin

It is believed that doxorubicin used in OSA treatment is as effective as cisplatin or carboplatin. One of the first researches on doxorubicin’s effectiveness was conducted in 1995. Berg and associates (1995) compared the results of 35 dogs with appendicular OSA treated with 5 doses of doxorubicin (30 mg/m2 of body surface, i.v., every 2 weeks) and limb amputation (after second or third dose) with a historical control group of 162 dogs who were treated with amputation alone. The median survival time for dogs receiving adjunctive therapy was 366 days, which was significantly longer than for the control group (138 days). Similar results were presented by Moore et al. (Moore et al. 2007), whose study included 303 dogs with appendicular OSA. The way of doxorubicin administration was similar to the one in the previous report. Doxorubicin demonstrated efficacy in the slowing of metastasis in dogs with appendicular OSA with a 1-, 2-, and 3-year median survival time of 35, 17, and 9 % respectively (Moore et al. 2007). The results obtained are similar to those with carboplatin as the adjunctive method of treatment, which indicates that both drugs may be used to prolong patients’ lives, however, neither of them inhibits metastasis.

#### Alternative treatment

Alternative chemotherapy protocols include using lobaplatin or ifosfamide. Salvage treatment with ifosfamide was evaluated in the group of 19 dogs with OSA and previously treated with standard chemotherapy. Median ifosfamide dosage was 375 mg/m2 administered on average 2 times. The finding indicated that ifosfamide was well tolerated but has minor anti-tumor activity (Batschinski et al. 2012). Promising results were described by Kirpensteijn et al. (2002), who examined the efficiency of lobaplatin on the group of 28 dogs with OSA. Dogs were treated with surgical resection of undertaken limb and adjuvant lobaplatin chemotherapy at a dose of 35 mg/m2 i.v. once every 3 weeks, for a maximum of 4 doses. Compared to historic controls treated with surgery alone, the results suggest that lobaplatin prolonged the disease free interval and survival time in dogs with OSA. More than 20 % of dogs achieved a 1 year disease free interval and more than 30 % of dogs reached a 1 year survival time (Kirpensteijn et al. 2002).

#### Combined chemotherapy

Another attempt to improve chemotherapy’s effectiveness was to compare the effects of two cytostatic drugs given in an alternating schedule. The efficiency of alternating the administration of cisplatin and doxorubicin after amputation was evaluated. 38 dogs treated with combined therapy after amputation had a significantly longer survival time than dogs that were treated with amputation alone, yet the result was still similar to the one achieved during monotherapy which involves carboplatin or doxorubicin (Mauldin et al. 1988; Chun et al. 2000). Moreover, the results of subsequent studies confirm that a disease-free interval and survival time are close to those reported for single-agent protocols (Kent et al. 2004; Bacon et al. 2008). Similar conclusions were presented by Selmic et al. (Selmic et al. 2014), who performed a retrospective cohort study which included 470 dogs with appendicular OSA. They compare their median survival time and adverse effects after therapy with surgery and carboplatin, doxorubicin or both of them (using 5 different protocols). The results achieved demonstrate that combining the two drugs does not increase the median survival time in comparison to monotherapy. However, lower adverse effects were observed in those patients. On the other hand, Lane et al. (2012) and Skorupski et al. (2013) indicate that dogs with appendicular OSA receiving carboplatin alone had significantly longer disease-free intervals than dogs receiving carboplatin and doxorubicin in an alternating schedule. To sum up, there is no clear evidence if combined chemotherapy is more efficient than single agent therapy, however, it may reduce negative side effects, which might indicate that using multiple drugs in a long lasting therapy increases the quality of patients’ lives.

#### Experimental treatment

In order to increase efficacy of OSA treatment and reduce the metastasis ability scientists performed many studies in which they modified standard chemotherapy treatment by adding different substances, such as: pamidronate (a nitrogen containing bisphosphonate, antiosteoporosis drug, which was found to show high cytotoxicity against osteosarcoma cell lines), gemcitabine (nucleoside analog used as chemotherapeutic agent), BAY 12–9566 (a matrix metalloproteinase inhibitor that shows a possibility to inhibit metastasis ability of tumor cells), suramin (a polysulfonated naphylurea which in vitro increases the tumor’s sensitivity to chemotherapeutic agents) or liposome-encapsulated muramyl tripeptide (which has been shown to regress spontaneous metastasis by activating macrophages). Unfortunately, the results showed that the addition of pamidronate to carboplatin chemotherapy for treatment of canine OSA, despite being safe, does not impact the efficacy of standard treatment (Kozicki et al. 2013). Also the results achieved by enriching carboplatin monotherapy with gemcitabine were comparable to those reported for carboplatin alone, which did not improve the outcome (McMahon et al. 2011). Another study was performed on 303 dogs to check whether adding BAY 12–9566 can improve OSA treatment based on doxorubicin followed by limb amputation. Treatment with BAY 12–9566 did not influence the survival time. The median survival time in both groups was less than 8 months (Moore et al. 2007). Authors evaluated the combination of noncytotoxic suramin and doxorubicin after amputation in dogs with OSA. In conclusion of this experiment similar results to monotherapy median disease free time was achieved (Alvarez et al. 2014). Also Kurzman and associates (1995) showed that benefits of adding Liposome-encapsulated Muramyl Tripeptyde to standard chemotherapy treatment (with cisplatin) and surgery is time. They showed that there is no survival advantage of administering Liposome-encapsulated Muramyl Tripeptyde concurrently with cisplatin chemotherapy and surgery, while the addition of Liposome-encapsulated Muramyl Tripeptide following cisplatin treatment significantly increased the median survival time (14,4 months) as opposed to other groups - treated with surgery, cisplatin and liposomes alone (9,8 months). This dogs had also a significantly longer metastasis-free interval (*p* < 0.035) compared to dogs given placebo liposomes. The results obtained by Kurzman et al. (1995) show that Liposome-encapsulated Muramyl Tripeptide has significant antitumor activity when administered alone, but this effect is not observed when giving concurrently with chemotherapeutic agent as cisplatin.

#### New therapeutic approach to overcome multidrug resistance

One of the most important cause of ineffective chemotherapy treatment of OSA is due to multidrug resistance (MDR). MDR in cancers is frequently associated with the overexpression and higher activity of efflux pumps (mainly p glycoprotein – PGP) that prevents intracellular accumulation of the drugs in cancer cells resulting in obtaining too low concentration of the drug inside the cell. PGP is a gene product of multidrug resistance protein 1 gene (MDR1). As a result silencing MDR1 mRNA expression is a new approach for overcoming MDR. On the other hand, some nanoparticles due to their nanoscopic size (lower than 100 × 10^−9^ m) have the ability to bypass MDR entering the neoplastic cells through endocytosis. Susa et al. (Susa et al. 2010) in a pilot in vitro studies showed that both doxorubicin and siRNA encapsulated in liposome-based dextran nanoparticles cause the simultaneous suppression of drug efflux pumps and have higher cytotoxic effect both on drug sensitive and drug resistance OSA cells. Kimura et al. (2013) and Sha et al. (Sha et al. 2013) presented in vitro studies with fucoidan nanoparticles and dioxide nanoparticles on human osteosarcoma cell lines, which suggest that they may be a potent new therapeutic agents to treat primary tumors as well as to minimize or prevent the reccurence of OSA. However, in vivo studies should be performed to confirm such hypothesis.

Until now, in veterinary medicine only a few studies including nanoparticles for OSA treatment have been performed, e.g. including the use of STEALTH liposome-encapsulated cisplatin. STEALTH liposomes as nanocarriers should slowly release the antracyclin drug in the acidic environment of the neoplastic tissue and should enable to reach higher concentration of the drug in tumor tissue according to retention and permeability effect. As a result, Vail et al. (Vail et al. 2002) compared STEALTH liposome-encapsulated cisplatin therapy (SPI-77) with standard carboplatin therapy in dogs with OSA. Dogs were treated with SPI-77 in 350 mg/m2 dosage i.v. 3-times every 3 weeks. Carboplatin was given in standard therapy (300 mg/m2 i.v. every week for 4 treatments). However, the results obtained were unsatisfactory as dogs treated with carboplatin alone achieved similar disease free-survival and overall survival compared to dogs treated with SPI-77 (disease free survival time was 156 and 123 days, respectively (*p* = 0.19). (Vail et al. 2002).

### Radiotherapy

Radiation therapy is considered to be a palliative method of treatment. The intent is to provide pain relief and prolong patients’ lives. However, it is not easily accessible and requires a general anesthesia of the patient. Studies performed by Oblak et al. (Oblak et al. 2012) proved that combined therapy including surgery, chemotherapy and radiotherapy is nowadays the most effective way of treatment. Fifty dogs were included in the study and median survival times between those that received palliative radiation therapy alone, and in combination with chemotherapy, pamidronate, or both were compared. Median survival times were the longest for dogs receiving radiotherapy together with chemotherapy (307 days) and the shortest in dogs receiving radiotherapy and pamidronate (69 days). Chemotherapy in addition to radiotherapy gave satisfying results, while enriching radiotherapy with pamidronate was ineffective (Oblak et al. 2012).

## Conclusions

Canine osteosarcoma is a highly malignant bone tumor in dogs with a high metastasis ability. Nowadays, combining surgery with single agent chemotherapy and radiotherapy seems to be the most effective method of OSA treatment. However, reducing the high metastasis ability and enhancing antitumor activity of cytostatic drugs having minimal negative side effects is still a challenge for veterinary practitioners. New approaches of OSA treatment including the use of nanoparticles are currently under investigation. The results of in vitro studies with fucoidan nanoparticles or dioxide nanoparticles on human osteosarcoma cell lines suggest that they may be a potent new therapeutic agents to treat primary tumors as well as to minimize or prevent the recurrence of OSA . Doxorubicin and siRNA encapsulated in liposome-based dextran nanoparticle could potentially be utilized to develop novel therapies. Many such therapies used in human medicine aimed at stopping metastatic disease could be helpful in dogs with OSA, however, further in vitro and in vivo studies in veterinary medicine are needed.
